# Students’ “teleological misconceptions” in evolution education: why the underlying design stance, not teleology per se, is the problem

**DOI:** 10.1186/s12052-019-0116-z

**Published:** 2020-01-09

**Authors:** Kostas Kampourakis

**Affiliations:** Section of Biology and IUFE, University of Geneva, Geneva, Switzerland

**Keywords:** Teleology, Evolution education, Selection teleology, Design teleology, Design stance, Consequence etiology, Functions, Natural selection, Design

## Abstract

Teleology, explaining the existence of a feature on the basis of what it does, is usually considered as an obstacle or misconception in evolution education. Researchers often use the adjective “teleological” to refer to students’ misconceptions about purpose and design in nature. However, this can be misleading. In this essay, I explain that teleology is an inherent feature of explanations based on natural selection and that, therefore, teleological explanations are not inherently wrong. The problem we might rather address in evolution education is not teleology per se but the underlying “design stance”. With this I do not refer to creationism/intelligent design, and to the inference to a creator from the observation of the apparent design in nature (often described as the argument from design). Rather, the design stance refers to the intuitive perception of design in nature in the first place, which seems to be prevalent and independent from religiosity in young ages. What matters in evolution education is not whether an explanation is teleological but rather the underlying consequence etiology: whether a trait whose presence is explained in teleological terms exists because of its selection for its positive consequences for its bearers, or because it was intentionally designed, or simply needed, for this purpose. In the former case, the respective teleological explanation is scientifically legitimate, whereas in the latter case it is not. What then should be investigated in evolution education is not whether students provide teleological explanations, but which consequence etiologies these explanations rely upon. Addressing the design stance underlying students’ teleological explanations could be a main aim of evolution education.

## “Why?” questions

Let us begin with a simple question: “Why do we have a heart?”. If you ask students, but also scientists, this question, a likely answer to receive is: “In order to pump blood.” We usually ask “Why?” questions in our attempt to explain a phenomenon, in other words in order to identify its causes. However, does the phrase “In order to pump blood” causally explain the fact that we have a heart? This is a conceptually tricky issue that teachers and educators need to approach thoughtfully in order to make students understand the issues at stake. Let this be our guiding question in exploring what teleology is. The question I therefore intend to answer in this essay is the following: Is the explanation “We have a heart in order to pump blood” a scientifically legitimate one for the presence of a heart?

Generally speaking a “Why?” question can be answered with reference to three kinds of causes (based on Mayr 1961; Ariew 2003): ultimate causes, proximate causes, and final causes. Ultimate causes are to be found in the distant past and relate to the evolution of a species. Thus, an explanation based on ultimate causes answering the question “Why do we have a heart?” could be “Because this organ provided an advantage to its bearers and there was selection for it, which resulted in this organ becoming prevalent in our ancestors”. This is an explanation of the fact that we have hearts as the outcome of a selective advantage of this organ for our ancestors. Proximate causes are to be found in the recent past and relate to the development of individuals within a species. Thus, an explanation based on proximate causes answering the question “Why do we have a heart?” could be “Because the cells in that area of the body of the individual were differentiated to become heart muscle.” This is an explanation of the fact that we have a heart as the outcome of a developmental process that resulted in the formation of this organ in one’s body. Both explanations based on ultimate and proximate causes are backward-looking, and refer to evolutionary and developmental causes and processes, respectively. Therefore, there exist both evolutionary and developmental explanations for the existence of hearts.

However, there is a third type of causal explanation that is based on final causes and that is forward-looking, as it refers to a specific contribution that this organ makes. Given that a function can be defined as an effect that makes a specific contribution, and that pumping blood is a contribution that the heart makes to our body, we can consider pumping blood as the function of the heart. Therefore, the question “Why do we have a heart?” can also be given the answer “In order to pump blood”. This is a teleological explanation for the existence of the heart; according to this, the heart exists for performing a function, which can be considered as a final cause because it is the reason for which the heart exists. This kind of teleological explanations has been found to be prevalent among students of all ages (see e.g. Kelemen 2012). Table 1 summarizes the features of the causal explanations for the existence of a heart.

**Table 1:** The main types of causal explanations and their features

“Why?” question	Causal explanation	Temporal dimension	Level of reference	Causes	Type of causal explanation
“Why do we have a heart?”	Because there was selection for this organ that thus became prevalent in our ancestors	Backward-looking	Population	Ultimate causes (evolution)	Evolutionary explanation
	Because the cells in that area of the body of the individual were differentiated to become heart muscle	Backward-looking	Individual	Proximate causes (development)	Developmental explanation
	In order to pump blood	Forward-looking	Population or individual	Final causes (function)	Teleological explanation

Many science educators, myself included (see e.g. Kampourakis and Zogza 2008, 2009), have used the adjective “teleological” to describe students’ misconceptions. However, this can be misleading. To understand why, we need to look at the nature of teleological explanations in some detail. Students usually describe the function of an organ or another part of the body by providing a teleological explanation for its existence. For instance, if a student states that eagles have wings in order to fly, this is a teleological explanation for the existence of wings that relies on the function that the wings perform (in this case, the effect of their movement that contributes to flight). Whether or not the parts of organisms perform functions is a question that has been debated among philosophers of biology, but in this essay I side with those who have argued that they do (e.g. van Hateren 2017, Weber 2017). Of course, not all of our body parts have functions; but some do perform functions that are important for the respective organism. The question then becomes: is the reference to the function of the heart a sufficient grounding for explaining its existence? In this essay, I argue that the problem in biology education is not the use of teleological/functional explanations; rather, the problem lies in the underlying etiology that relates to how these functions came to be. The issue here is that the teleological explanation that we have a heart in order to pump blood can actually be a scientifically legitimate explanation for the presence of the heart. Let us now see why.

## The nature of teleological explanations

In general, teleological explanations are those in which a phenomenon is explained in terms of a final end (telos) to which it contributes. Teleological explanations are characterized by expressions such as “… in order to ….”, “… for the sake of…”, “… so that …” etc., and they go back to the philosophies of Plato and Aristotle, even though the term was coined in 1728 by the philosopher Christian Wolff (Lennox 1992; Ariew 2007; Walsh 2008). In *Timaeus*, Plato considered the universe as the artifact of a Divine Craftsman, the Demiurge. He considered the universe as a logical, living entity possessing a soul that was the primary cause of any transformation. This soul controlled everything through the final causes that determined every action in which it was involved, thus imposing control on any chance events (Κάλφας 1995, pp 85–90). For Plato, the final cause of the creation of the universe was the transfusion of the soul of the Demiurge into his artifact, which could be achieved by the imposition of order over disorder (Κάλφας 1995, pp 69–70). This process had to take into account the actions of Need, the mythical equivalent of the properties of the structure of matter, which seemed to impose constraints to the work of the Demiurge. Plato thus recognized two types of causes: the divine (final) and the necessary (mechanistic), and thought that they were interdependent and not in conflict (Κάλφας 1995, p 283). Consequently, the universe was an artifact that resulted from the purposeful and rational action of the Demiurge who had dominated over the irrational Need (Κάλφας 1995, p 92). This idea eventually perceives the world as “unnatural”, as it is not the product of natural processes but of a wise craftsman (Lennox 2001, p 281).

Aristotle was a student of Plato who, contrary to his teacher, attempted to find natural causes within the organisms, rather than beyond them. He thought that there were four causes acting in nature and that knowledge could be gained through their understanding. These causes were the Efficient cause, the Material cause or matter, the Formal cause or form and the Final cause; Aristotle considered all four of them as necessary for explanations (Κάλφας 1999, pp 116–117). Matter referred not only to the material a body was made of, but also to any circumstance required to make this happen; whereas Form referred to the internal structure and not only to shape of the body (Κάλφας 1999, p 201). Aristotle thought that final causes served the maintenance of the organism. In other words, the final cause for the existence of an organ would be its usefulness to the organism that possessed it. Contrary to Plato, who assumed intentional design, Aristotle thought that organisms acquired some features simply because they were functionally useful to their life (Lennox 1992, 2001). For Aristotle the teleological approach was the main approach to understanding biological phenomena. In many cases this approach actually helped him identify functions that would not have been noticed in a solely descriptive approach. Aristotelian teleological explanations are therefore “natural”: whatever is explained in teleological terms exists because it has positive consequences for its possessor, without any intention or design.

To summarize: On the one hand, teleological explanations can be based on intentional design, that is, one can state that a feature exists because it was intentionally created for a purpose. On the other hand, teleological explanations can be based on functionality, that is, one can state that a feature exists in order to perform a function that is useful for the whole to which this feature is belongs. Design-based explanations are legitimate for artifacts, which are designed and created for an intended use. However, they are scientifically illegitimate for organisms because there is evidence that they are not designed as they contain many useless or malfunctioning features. In contrast, function-based teleological explanations are scientifically legitimate for organisms because our organs and several (but not all) body parts do perform functions that are useful to the organisms that possess them. Therefore, the first point to note is that teleology that relates to function is legitimate in biology.

Let us now consider functional explanations. The explanation for the presence of an organ on the basis of the consequences of its existence, which is its function, is described as a consequence etiological approach to function, or *etiological analysis*, as it is based on processes that presuppose consequence etiologies. This is a historical view of functions, according to which a feature exists because of the consequences that it has, or simply put because of what it does. In other words, if we say that the function of X is F, this means that X is there because it does F and that F is a consequence (or result) of X’s being there (Wright 1973). This view has been contrasted to an ahistorical view described as *functional analysis* (Cummins 1975). In particular, it has been argued that to explain the existence of a trait in terms of its function provides an inadequate view of the evolutionary process (Cummins 2002). According to this view, biological traits exist not because of their functions but because of their developmental histories. Whether or not a trait has a function and what that function happens to be is independent of whether the trait was selected for it. To explain selection, one must look not at the function of a trait but on how well the several varieties of a trait are functioning, because selection presupposes the existence of such variation. Such explanations “either run into the fact, fatal to classical teleology, that the crucial details of evolutionary (or ontogenic) development predate anything with the function that is supposed to do the explaining, or they founder on the fact that competing traits in selection scenarios typically have the same function. Things don’t evolve because of their functions any more than they develop because of their functions” (Cummins 2002, p 169).

More recently, philosophers have suggested that these two views should be integrated with each other. On the one hand functional analysis focuses on the identification of current causal contributions of traits in complex physiological and other processes. On the other hand, etiological analysis focuses on the origin of functions through selective processes, with functions making causal contributions as a result of older selection pressures. Functional analysis on its own can be quite liberal as it might explain any complex system as performing a function e.g. that a function of a particular arrangement of rocks is to contribute to the widening of a river delta or that the function of particular mutations is to promote the formation of tumors. At the same time, the etiological analysis may not be always applicable as there are traits that perform functions even though they have not been selected for these functions. However, when this is the case the etiological analysis can provide useful information for the origin of a trait, which cannot be obtained through functional analysis (see Kitcher 1993; but also Godfrey-Smith 1993).

To summarize: On the one hand, functions can be historical, that is, something that performs a function can be said to exist because of the benefits that this function confers to its bearers, and as a result it has been favored by selection because of this. On the other hand, functions can be ahistorical, that is, something that performs a function cannot be said to exist because of the benefits that this function confers to its bearers, as it does not have to have been favored by selection because of this. It is indeed the case that some functions exist because the respective features have been and/or currently are being favored by selection; but it is also the case that some features may perform genuine functions without any kind of selection going on. The second point I want to make is that function-based teleology is meaningful only if it is historical, in other words if we assume a selection history behind it. In other word, only if a feature has been selected for the function it performs, can it be said to exist in order to perform that.

A last point to consider is the difference between functional explanations for organisms and functional explanations for artifacts. Given that artifacts are designed with an intended effect or use in mind, we can state that an artifact has a specific function only if an agent has had the intention for the particular artifact to perform this function. In other words, the function of an artifact is whatever effect its maker intended it to have. This can be described with the following proposition: The function of artifact A is F if an agent X intended A to perform F. When it comes to organisms, however, there is no agent to whom an intention for an organ to perform a function can be ascribed. But there is a sense in which an agent X can select an artifact A in order to perform a function F. In a similar sense, a trait T can be selected for performing an effect—which can be considered to be its function if this effect contributes to the survival and reproduction of its bearers. This can be described as the selected effects account of biological function and the respective proposition can take the following form: The function of trait T is F if it has been (naturally) selected to perform F. Both of these accounts are etiological because in both cases the function is based on the artifact’s and the trait’s history (based on Lewens 2004, pp 89–91).

Considering all the above, we can distinguish between two types of teleological explanations. On the one hand, there exist teleological explanations that are based on design. In this case, something exists because of its consequences that contribute to the fulfillment of an agent’s intention, external to the organism, to achieve a goal. Thus, intentional design is assumed. In other words, *the cause of the existence of a particular feature is the external agent’s intention to fulfil this goal*. To illustrate this, imagine trying to explain why a population of beetles living in a mostly brown habitat all have brown color, even though the initial population some generations ago consisted of both green and brown beetles. An explanation based on design might state that an external agent (Nature, God, or whatever) had the intention to preserve this population of beetles and so caused mutations that made them change from green to brown, in order for them to be able to conceal themselves and avoid predations from birds. This kind of teleology can be described as *design* teleology. In this case, “design” refers to the intention of an external agent. There is also another version of design teleology, in which the intention is internal, in the sense that it refers to the intention of the organisms themselves to fulfill their needs. So, design teleology can be either intention-based (depending on the intentions of an external agent, or simply external) or need-based (depending on the needs of the organism itself, or simply internal).

On the other hand, there exist teleological explanations that are based on natural processes. In this case, something exists because of its consequences that contribute to the well-being of its possessor, without any assumption of intentional design. In the beetle example, the explanation would therefore be that from the initial population of brown and green beetles, it was only some brown ones that survived and reproduced because the green ones were gradually eliminated due to predation by birds. In other words, *the cause of the existence of the brown color is the advantage it conferred to its bearers*. There was selection for brown color, because it conferred a survival advantage to its bearers and this is why it can be now considered to exist for this purpose. However, this is a purpose fulfilled through a natural selection process. This kind of teleology can be described as *selection* teleology (Lennox and Kampourakis 2013; see also Lombrozo and Carey 2006). Let us consider this in some more detail. The description of the selection for brown color can be rewritten as follows (see Lennox 1993; Lennox and Kampourakis 2013):

- Brown color is present in the population of beetles living in the brown environment.
- Brown color provides concealment to its bearers in the brown environment.
- Concealment is advantageous as brown beetles avoid predators.
- Therefore, brown color would be selectively favored in the population of beetles.
- Therefore, concealment is the cause of the presence of brown color in the population of beetles.

### This can also take the following more general form

- Trait V (brown color) is present in population P (beetles).
- Trait V (brown color) has effect E (concealment).
- Effect E (concealment) is advantageous (avoid predators) to its bearers in population P.
- Therefore, trait V (brown color) in population P would be selectively favored.
- Therefore, effect E (concealment) is the cause of trait V’s (brown color) presence in population P.

Because the effect E is the cause of trait V’s presence in population P, we can legitimately state that V exists in order to do E. This is a robust form of teleology. The main features of the three kinds of teleology are summarized in Table 2.

**Table 2:** The main features of design and selection teleology

Types of teleology	Consequence etiology	Assumption of design	Examples
Design teleology (external)	Something exists because of its consequences that contribute to the fulfillment of an external agent’s intention to achieve a goal	Yes (it is explicit as there is reference to the intentions of an external agent)	Green beetles mutated to become brown in order to conceal themselves, thus fulfilling the intention of an external agent (such as Nature, or God)
Design teleology (internal)	Something exists because of its consequences that fulfill the intentions/needs of its possessor	Yes (it is implicit as there is reference to the intentions/needs of the organism itself)	Green beetles mutated to become brown in order to conceal themselves, thus fulfilling their intentions/needs
Selection teleology	Something exists because of its consequences that contribute to the well-being of its possessor, and is thus favored by natural selection	No	Brown beetles had a concealment advantage compared to green beetles, which eventually died out due to predation, and thus only brown beetles survived and reproduced

A note of caution is necessary here. Teleological explanations based on functions are legitimate when they are causally justified. In other words, functions can be legitimately used in explanations only when they were also causes of whatever is being explained. For instance, in explaining how a population of green and brown beetles evolved to a population of brown beetles that are well-concealed in the brown environment in which they live, we can mention both the genes related to the brown color and predation of the less well concealed individuals as causal factors. But which of the two causal factors is the cause that made the difference? One way to decide is to see which one is likely to be useful in prediction by making a difference in future cases. In this case, it is the function of brown color in concealment that can have a predictive value and be expected to make a difference in future cases. This entails that functional–teleological explanations should be restricted to those cases where the function not only had a causal influence, but did so through a causal process that conforms to a predictable pattern (see Lombrozo 2006; Lombrozo and Carey 2006).

Even though it is true that not all functions are the outcome of selection, for the purposes of evolution education it might be useful to assume that this is the case, rather than leave students intuitively attribute functions to design. In other words, I argue that in order to refrain from having students intuitively use design teleology in their explanations, it is preferable to apply the historical—etiological view of functions and promote the use of selection teleology instead. This does not entail an ultra-adaptationist view that natural selection can explain everything; it cannot, because other natural processes such as drift are also important. Rather, the point here is that—for educational purposes—selection-based explanations could be presented as more likely and more legitimate than design-based explanations. Of course, empirical research is required in order to conclude whether students can indeed understand the difference between e.g. design and selection teleology, and whether they can learn to construct selection-based teleological explanations.

From all the above, we can reach a main conclusion. Explanations based on natural selection are causal, because they rely on causes that relate to past events, and they exhibit a robust form of teleology where something exists because it was selected to do what it does, and so can be said to exist for doing it. Therefore, the teleological explanations that students give can be legitimate scientifically. If students state that we have a heart in order to pump blood, the problem is not teleology per se, but the underlying consequence etiology. In the case of selection teleology, natural selection does the explaining, and this results in a scientifically legitimate explanation. What is problematic is that in the case of design teleology, the explanation is based on what has been described as the design stance: our tendency to perceive purpose and design in the world (for an overview of this research, see Part I of Kampourakis 2018). Therefore, it is the design stance and not teleology that we need to address in evolution education. But before considering what could be done, it might be useful to better understand what the design stance is about.

## The design stance

It has long been shown that children provide teleological explanations from a very young age. In one study, it was investigated whether 7–8-year-old children provided teleological explanations for both organisms and artifacts. They were asked to choose between two possible explanations for why plants and emeralds were green: (1) they are green because this helps having more of them, or (2) they are green because they consist of tiny green parts. This is a difficult question because the latter explanation is actually correct for both plants and emeralds: plants are green, or have parts that are green, because they contain chloroplasts that are small, intracellular organelles filled with chlorophyll; whereas emeralds have green color because they contain traces of chromium and vanadium. However, one might also argue that being green is an advantage for plants because chlorophyll makes photosynthesis possible, and so plants can transform energy and live. Even though the complete explanation of why being green helps plants exist can be considered as advanced for 7-year-olds to understand, most of them preferred that explanation for plants and not the physical one, which in contrast they mostly preferred for emeralds (Keil 1992, pp 129–130).

The distinction made above between a physical explanation (being green because of consisting of tiny green parts) and a teleological explanation (being green helps having more of them) reflects two different stances that have been described as the physical stance and the design stance, respectively. The physical stance is the use of whatever we know about physics (e.g., how objects fall to the ground) in order to make predictions or explanations. It generally works for all kinds of entities—organisms, artifacts, and nonliving natural objects. For instance, if I hold a plant, a watch, or an emerald and I suddenly release them, they will all fall to the ground. The design stance is a different strategy that relies on additional assumptions, which are that a specific object is designed and that it will operate according to that design. There is also a third one, the intentional stance, which can be considered as a subspecies of the design stance (Dennett 2013, Chap. 18).^1^ Therefore, we can simply make a distinction between the physical stance and the design stance. The question thus becomes whether we prefer to explain a particular feature on the basis of its physical properties or on the basis of the function that this feature seems to serve.

Here lies the problem: whereas we can make similar predictions for a plant, a watch, and an emerald using the physical stance, we cannot do the same using the design stance. If we drop any of these objects from a high building to the ground, they will all fall down and break. This can be explained by using physics: the gravitational force brought the objects to the ground in an accelerated motion, and when they touched it a force (which we could actually estimate) was exercised on them, breaking them into pieces. It is as simple as that, and there is absolutely no difference in making a prediction or an explanation about these objects using the physical stance. If we drop them from a high building, they will all break for the same reason. However, the design stance does not allow us to see these objects in the same way. An emerald that broke into two pieces may have now become two smaller emeralds. However, the watch and the plant will be a broken watch and a broken plant. The design stance makes us think of the plant in the same terms as the watch rather than the emerald. In other words, the design stance makes us see a natural object, the plant, as an artifact such as the watch rather than as another natural object, the emerald. The reason for this is that we perceive functions to exist in both the watch and the plant, but not in the emerald.

It is very important to note that two distinct and consecutive inferences can be made, based on the design stance. The first one is from a particular structure/function to the existence of design, e.g. from the internal arrangement of the parts of the watch to the idea that this arrangement serves a purpose. The second one is from the existence of design to the existence of an intentional and intelligent designer, e.g. from the purpose served by the arrangement of the parts of the watch, which is to tell the time, to the watchmaker who had the intention to create such an artifact. This is very important to keep in mind because this is what in my view makes evolution counter-intuitive, and also makes the design stance a major conceptual obstacle for understanding evolution. Religious belief is of course an important emotional obstacle. But it can be the case that people do not reject evolution only because it conflicts with their worldviews and religious beliefs; rather, it can be the case that they perceive design in organisms, and this perception fits better with their religious belief about the existence of an intelligent designer rather than with the idea of evolution via natural processes. There is actually ample research that shows that people express beliefs in purpose and design in nature independently of their religious background (for overviews, see Kampourakis 2014, Chapter 2; Kampourakis 2018, Part I).

Therefore, there are two distinct inferences to consider. The first one is the teleological inference, which stems from the perception of design in organisms, and the second one is the inference to the existence of a designer. *I argue that the first inference is not problematic, whereas the second inference is.* What therefore biology educators and teachers could do is first to explain to students that the first inference is correct; the next step would then be to explain to students that the second—scientifically legitimate—inference to make is the inference to natural selection and not to design. In other words, teleological explanations are acceptable, insofar as it is made clear that the underlying consequence etiology is selection-based and not designed-based (see Table 2). This distinction and the conceptual obstacles we should address are presented in Table 3.

**Table 3:** The structure of teleological explanations; the difference is in the underlying consequence etiology

	Observation	1st inference: Teleological inference	2nd inference: Consequence etiology
Misconception/illegitimate explanation	Organisms have structure A that performs function B	Structure A exists in order to perform function B	Structure A exists in order to perform function B because a designer intentionally designed it for this purpose (design teleology—external)
Misconception/illegitimate explanation	Organisms have structure A that performs function B	Structure A exists in order to perform function B	Structure A exists in order to perform function B because it is necessary to its bearers for their survival/reproduction (design teleology—internal)
Legitimate explanation	Organisms have structure A that performs function B	Structure A exists in order to perform function B	Structure A was selectively favored because the function B that it performs confers an advantage to its bearers for their survival/reproduction (selection teleology)

What Table 3 shows is that the problem are not students’ teleological inferences per se, but rather the underlying consequence etiologies, that is, whether teleology is based on design or on natural selection. Returning to our guiding question: Is the explanation “We have a heart in order to pump blood” a legitimate one for the presence of a heart? The answer is yes, but only insofar as there is explicit reference to evolutionary causes and processes, and the fact that organisms are not designed. In other words, the problem is not to say that we have a heart in order to pump blood, but to attribute it to design rather than to natural selection. The important implication of this then for science education is how to distinguish between design and selection teleology. To achieve this, it might be useful to consider the differences between artifacts, which by definition exhibit design teleology, and organisms, which do not.

## Organisms and artifacts

There exists an enormous body of research that shows that from a very young age children tend to provide teleological explanations for organisms and artifacts (summarized in Chapter 3 of Kampourakis 2014). Despite the differences in the details, an important finding is that even if children perceive animals as being different from artifacts, they do not necessarily perceive animal parts differently from artifact parts. For instance, in one study, children’s questions about function were more frequent for animal parts than for whole animals, and overall the number of questions about parts was similar for organisms and artifacts (Greif et al. 2006). Similarly, in another study, 4- and 5-year-old children were found to provide teleological explanations for both animal and artifact parts, while they also realized that parts of organisms are more probable to have some use or function compared to whole organisms (Kelemen 1999). This entails that we might tend to intuitively think of the parts of organisms in the same way that we think about the parts of artifacts: as designed for a function. This is plausible as for the past few thousand years humans have been growing up in artificial environments, surrounded by artifacts that were made with the intention to fulfil a goal. Therefore, as from very early in our life we become familiar with the use of artifacts, it might then simply be the case that we extrapolate our understanding of intended use and functions to the natural world, with which we are less familiar. In order to address this issue, it is very important to explain to children as early as possible the differences between organisms and artifacts, and especially the differences in how their parts that might perform a similar function came to exist.

Consider the wings of birds and airplanes. We might ask why birds and airplanes have wings, and it would be reasonable to state in both cases that they have wings in order to fly. However, one should also keep in mind that there is a major difference between them: airplanes are artifacts intentionally designed for a purpose, whereas birds are not. Because airplanes are designed in order to fly, they have wings that are always of an appropriate size so as to allow take off and flight. For instance, even though a Cessna airplane has smaller wings than an Airbus, in both cases the wings are long enough to facilitate take-off and flight. No rational aircraft builder would ever design an Airbus with the wings of a Cessna, or vice versa, because it would be impossible for either of these airplanes to take off and fly. A Cessna with the wings of an Airbus would be impossible to take off because the wings would be too heavy for its body to hold. An Airbus with the wings of a Cessna would also be impossible to take off because it would never reach the necessary aerodynamic conditions for taking off. Therefore, for any airplane we can legitimately say that it has wings in order to fly because it was intentionally and intelligently designed for this purpose. The situation is different for birds. All birds have wings, but not all of them use these for flying. We can say that eagles have wings in order to fly, but this is not the case for penguins that have relatively small wings for their size, and thus cannot fly. However, penguins use their wings for swimming, and they can actually swim very fast underwater. We can say that penguins have wings in order to swim. But then, ostriches also have wings but use them neither for flying nor for swimming. Therefore, all birds have wings, but not all birds use their wings in order to fly. This happens because birds are not artifacts and their wings were not intentionally designed for flying. Birds, like all organisms, have come to possess their features through evolution and are not intelligently designed.

There is thus a major difference between airplanes and birds, and more generally between organisms and artifacts. Teleological explanations for artifacts presuppose design, whereas teleological explanations for organisms presuppose natural processes, i.e., evolution. The crucial distinction here is that artifacts have particular features in order to perform some function as a consequence of their being designed for this purpose, whereas organisms have particular features in order to perform some function as a consequence of their being selected during evolution. In this sense, artifact teleology is external, whereas organism teleology is internal. The wings of airplanes and eventually airplanes themselves serve their human creators and their intentions. If artifacts possess some character for some purpose, this is a purpose external to them which has been set by their human creators. In contrast, the wings of birds serve (if they do so) their possessors (and probably their own intentions: find food, avoid predators, etc.). If organisms possess some features that seem to serve some purpose, e.g., eagles have wings for flying, what is actually happening is that flying is a consequence of having wings and other appropriate body parts that serves the organisms themselves and not some agent external to them. Thus, organism teleology is based on consequences without a presupposition of intentional design and so differs significantly from artifact teleology.

Another problem for evolution education is that students often conceptualize an internal, need-based teleology that nevertheless is also based on the idea of design. In this case, the design reflects the intentions of the organism itself to fulfil its needs. The need to acquire a specific feature therefore becomes the causal factor that is used to explain the existence of a feature. The feature exists because the organisms needs it and therefore has to have it (design teleology), and not because it has been selected for the advantage that it confers to its possessors (selection teleology). In the first case teleology is unnatural and design-based, whereas in the second case it is natural and selection-based. The main types and features of artifact and organism teleology are summarized in Table 4.

**Table 4:** The main features of selection and design teleology

Types of teleology	Consequence etiology	Assumption of design	Legitimate for artifacts or organisms	Example of misconception	Example of correct conception
Design teleology (external)	Something exists because of its consequences that contribute to the fulfillment of an external agent’s intention to achieve a goal	Yes	Artifacts only	Eagles have wings in order to fly because God/Nature designed them in such a way in order to be able to use them for flying	Airplanes have wings in order to fly because humans designed them in such a way so as to be able to use them for flying
Design teleology (internal)	Something exists because of its consequences that contribute to the fulfillment of its possessor’s intention to achieve a goal	Yes	Neither artifacts, nor organisms	Eagles have wings in order to fly because they needed them in order to be able to use them for flying	–
Selection teleology	Something exists because of its consequences that contribute to the well-being of its possessor	No	Organisms only	–	Eagles have wings in order to fly because this feature appeared in their ancestors, provided them with an advantage and was therefore maintained in their lineage by natural selection

The suggestion I would like to make based on all the above is that it might be useful for science educators and teachers to address the design stance during the teaching of evolution. By this, I do not mean addressing any explicit creationism beliefs that students might have—this is a different issue. Rather I refer to the intuitive thinking of the parts of organisms as designed for a function, goal or purpose. This view does not explicitly assume the existence of a conscious designer but nevertheless considers the features of organisms as having all those properties that the parts of a designed artifact would have. This is often found in students’ views that organisms have the features they need in order to survive in a particular environment. In other words, whereas students may not actually think that organisms are designed, they may in practice think of their parts as if they were designed. How this conception might be addressed is the topic of the next section.

## Addressing the design stance in evolution education

What we might do during the teaching of evolution is to explicitly address the design stance and bring students to a conceptual conflict situation where they will realize that the design-based explanations are insufficient. To do this, we could contrast two kinds of explanations, a design-based and a selection-based one (based on Kampourakis 2014, pp 89–96, but significantly modified and elaborated). There are several ways that this could be done. What I am suggesting here is a general scheme, rather than a specific way or activity to do this. How this can actually be done and whether it works well with students is of course something that requires empirical research in the future.

Let us begin with the teleological proposition:

- [T] Organisms O have trait A in order to perform function B.

As already explained in detail above, this proposition is not inherently wrong. In contrast, it is a legitimate proposition, and in fact it can also be a legitimate explanation for the existence of a particular feature. The important issue is what underlies such a proposition. If such a proposition stems from the design stance, then the design-based explanation would have the general form:

- [DT] Organisms O have trait A in order to perform function B, because organisms have the features that are necessary for their survival.

Whereas the selection-based explanation would have the general form:

- [ST] Organisms O have trait A in order to perform function B, because the latter confers an advantage; consequently, this trait has been selected for doing this and has been maintained in their lineage.

Imagine now that we apply explanations [DT] and [ST] to explain why dolphins and sharks have hydrodynamic shapes. This would produce the explanations presented in Table 5.

**Table 5:** Design-teleological and selection-teleological explanations for the features of sharks and dolphins

Question	Design teleology	Selection teleology
(1) Why do dolphins have hydrodynamic shapes?	[DT1] Dolphins have hydrodynamic shapes in order to swim fast underwater, because organisms have the features that are necessary for their survival	[ST1] Dolphins have hydrodynamic shapes in order to swim fast underwater, because the latter confers an advantage; consequently, this feature has been selected for doing this and has been maintained in their lineage
(2) Why do sharks have hydrodynamic shapes?	[DT2] Sharks have hydrodynamic shapes in order to swim fast underwater, because organisms have the features that are necessary for their survival	[ST2] Sharks have hydrodynamic shapes in order to swim fast underwater, because the latter confers an advantage; consequently, this trait has been selected for doing this and has been maintained in their lineage
(3) Why don’t dolphins have gills?	[DT3] Dolphins do not have gills, but have lungs in order to get more oxygen directly from the atmosphere, because organisms have the features that are necessary for their survival	[ST3] Dolphins do not have gills because this feature was not maintained in their lineage and because lungs evolved in their terrestrial ancestors
(4) Why do sharks have gills?	[DT4] Sharks have gills in order to breathe underwater, because organisms have the features that are necessary for their survival	[ST4] Sharks have gills in order to breathe underwater, because the latter confers an advantage; consequently, this trait has been selected for doing this and has been maintained in their lineage

Obviously, proposition DT1 is compatible with DT2 and ST1 is compatible with ST2. However, propositions DT3 and DT4 are incompatible. Why would two organisms, which both live underwater, have different organs for breathing had they been designed (or, more generally, were they formed in a way that satisfies their needs)? On the other hand, propositions ST3 and ST4 are compatible with each other. So, when the explanatory scheme ST is used, it produces propositions ST1 to ST4 which are all compatible with each other. In contrast, when the explanatory scheme DT is used, some of the propositions produced (in particular propositions DT3 and DT4) are logically incompatible. Therefore, the design stance is simply explanatorily insufficient.

A simple way to illustrate this takes the form of the following narrative (which I once watched during a documentary film): A big gray whale was swimming in the ocean, close to the surface, with its newborn that was barely the size of a big dolphin. The newborn was swimming very close to its mother’s body. If you ask any student why these animals have hydrodynamic shapes, they will immediately reply that they have them in order to swim fast under water. So far so good. Then, suddenly, two orcas, which are also mammals like the whales, approached the mother whale and the newborn, and tried to separate them. The orcas did not get very close to the mother whale as it could hit them hard, and so tried for a long time to separate her and the newborn. Eventually they succeeded, and then they repeatedly pushed the newborn into the sea until it drowned. But this would not have happened if gray whales had gills. The question that one can ask students then is why don’t whales have gills? The answer is simply that organisms may have particular features in order to perform a function, but they have neither optimal characters, nor ones that fulfill every possible need. There are indeed some features that exist in order to perform a function and they exist because natural selection has favored the survival and reproduction of their bearers. Organisms do not have all the features that they need in order to live in a particular environment. This is why dolphins and sharks, compared above, differ significantly in many characters, even though they live in similar environments. Dolphins have forelimbs, whereas sharks have fins; dolphins have mammary glands whereas sharks do not; dolphins have lungs whereas sharks have gills; dolphins have blowholes whereas sharks do not; and many more.

Why would two kinds of organisms that live in the same environment be so different from each other? The answer is simple: because they have evolved, and they were not designed.

## Conclusions

The adjective “teleological” is often used to describe students’ misconceptions about evolution in the literature. However, what is wrong in these misconceptions, is not teleology per se; the idea that a feature may exist in order to perform a function is not necessarily wrong, because if a feature has been selected for the function that it performs, then this function is the reason that it exists and this is a robust form of teleology. What is wrong is, rather, the reason for which this function came to be. Insofar as a feature exists because of a selection for it, this is a selection teleology based on natural processes, which is legitimate. What is problematic is the attribution of this function to a design teleology, that is, to argue that a feature exists because of the intentions of an external agent, or because of the needs of the organism itself. It is therefore important for biology educators and teachers to realize that it is legitimate to state, e.g., that humans have a heart in order to pump blood. What they should therefore address is not the statement itself, but the underlying consequence etiology, or why students make this statement. The ultimate goal of teaching would be to explain to students that functions are the outcome of natural processes, such as selection, and not of the fulfilment of any intentions or needs.

## Data Availability

Not applicable.

## Abbreviations

ST: selection teleology
DT: design teleology

## Footnotes

1 The reason for this is that the intention of the designer can be considered as a property inherent in the design, exactly because the properties of the designed object reflect the intentions of its designer. A chair and a table both have for legs, but are designed to serve different purposes: chairs are for sitting and tables are for putting our meal upon them. It would be unusual, indeed weird, to sit on a table and put our meal on the chair and start eating. The reason for this is that the intended use of artifacts, what they were made for, is evident in their design and use, or in other words the intention of the designer is inherent in the design of artifacts.
